# Determinants of Harem Size in a Polygynous Primate: Reproductive Success and Social Benefits

**DOI:** 10.3390/ani11102915

**Published:** 2021-10-09

**Authors:** Wancai Xia, Cyril C. Grueter, Baoping Ren, Dejun Zhang, Xiaoxia Yuan, Dayong Li

**Affiliations:** 1 Key Laboratory of Southwest China Wildlife Resources Conservation (Ministry of Education), China West Normal University, Nanchong 637009, China; xiawancai2013@163.com; 2 Institute of Rare Animals and Plants, China West Normal University, Nanchong 637009, China; 3 School of Human Sciences, University of Western Australia, Perth, WA 6009, Australia; cyril.grueter@uwa.edu.au; 4 Centre for Evolutionary Biology, School of Biological Sciences, The University of Western Australia, Perth, WA 6009, Australia; 5 International Centre of Biodiversity and Primate Conservation, Dali University, Dali 671003, China; 6 Key Laboratory of Animal Ecology and Conservation Biology, Institute of Zoology, Chinese Academy of Sciences, Beijing 100101, China; renbp@163.com; 7 School of Basic Medical Sciences, North Sichuan Medical College, Nanchong 637000, China; 1989615zdj@163.com (D.Z.); yxx19890830@163.com (X.Y.)

**Keywords:** harem size, one-male unit, snub-nosed monkey, reproductive success, grooming

## Abstract

**Simple Summary:**

The size of primate ‘harems’ varies considerably, both inter- and intra-specifically. Previous studies have shown that females prefer high-quality males and that high-quality males are superior in inter-male competition, leading to them having a larger harem size. Based on eleven years of observations of Yunnan snub-nosed monkeys (*Rhinopithecus bieti*), we documented longitudinal stability in the distribution of harem sizes between 2010 and 2020. These demographic properties are the outcome of male and female social investment decisions that affect their reproductive performance and success. Male reproductive success was positively related to harem size, while constraints on individual social benefits and social investments limited harem size. Our findings advance our understanding of the socioecological determinants of harem size variation in polygynous primates.

**Abstract:**

We used long-term data on the variation in harem size in Yunnan snub-nosed monkeys to research the effects of harem size on reproductive success and the ratio of grooming received to given (RGRG). The results suggest that harem holders derive reproductive benefits commensurate with harem size, whereas the females’ reproductive success is unaffected by harem size. Males of larger harems groomed less and had higher RGRG than males of smaller harems. In the case of females, grooming given increased, and RGRG decreased with an increase in harem size. The males’ reproductive success seems to be a driver of harem size maximization. From the females’ perspective, dwindling social benefits appear to set the upper limit for harem enlargement. We also showed that males of monogamous units (‘single-female harems’) invested more into grooming their female, presumably to prevent unit disintegration and loss of mating privileges.

## 1. Introduction

Polygyny is a common mating system in mammals and birds and comes in two main forms [1,2]: males either guard high-quality resources to attract females (resource defense polygyny), or they directly control access to a group of females clumped in space (female defense polygyny) [1,3,4,5]. Variance in male reproductive success is pronounced in a female defense polygyny system with sexual dimorphism and other secondary sexual traits (badges of status and ornaments) affecting male mating success [6,7,8].

The size of a harem reflects the outcome between optimizing individual reproductive strategies and minimizing costs [2]. A male’s reproductive success is limited primarily by the number of females that he can inseminate, so males with larger harems accrue higher reproductive success [9,10]. Co-resident females experience competition over resources and the sexual attention of males [11,12,13,14]. The high energy requirements of female mammals during pregnancy and lactation mean that their reproductive success rate is often limited by access to resources [15,16,17,18]. The intensified resource competition resulting from an increase in group size can impose physiological and social stress on females, thereby depressing fertility [2,19,20]. The other type of female–female competition is competition over breeding opportunities, which can manifest itself in the form of reproductive interference [21], aggression [22], the eviction of rivals [23], and physiological suppression of subordinate females [24]. There will thus be selective pressures to maintain the group size at a healthy level. Mechanistically, the main avenue through which this homeostasis is achieved is via dispersal to smaller groups [25,26,27,28]. Besides reducing competition, females may also leave their harems to improve their reproductive prospects, e.g., by seeking a high-quality male [29].

Most primates live in permanent heterosexual social groups within which they use grooming as an affiliative social tool [30,31,32]. Grooming can fulfil a hygienic function, providing tangible benefits in the form of ectoparasite and dirt removal [33,34,35]. Grooming can also have a derived social function; it can be used to establish and reinforce social bonds [32] and to maintain group cohesion [30,32]; see also [36,37]. Grooming behavior can be viewed as a longer-term investment aimed at attaining certain benefits [38], and its prevalence should fluctuate with group size [30,39]. When the group size increases, individuals need to devote more investment to social grooming to maintain social relationships with a larger number of individuals or increase grooming towards valuable partners [32]. Biological market theory holds that individuals could exchange grooming for in-kind reciprocal benefits [40] or other commodities/services, based on economic laws of supply and demand [41,42,43]. Some of these commodities include coalitional support [44,45], food [46,47], tolerance at food sources [48,49], mating opportunities [50], and infant handling [50,51]. In a group-guarding polygynous mating system where the resident male monopolizes mating rights and has priority of access to food, females may need to invest into grooming the male to gain access to his reproductive services [43,52]. Females may groom the resident male to form good social bonds [43,52]. When reproductive competition is intense, females may resort to grooming the resident male as an incentive for the male to offer his protective services. Females may also benefit from social investment into co-resident females to be granted tolerance at resources [43]. With increasing harem/group size and corresponding intra-sexual competition, females will have to raise their investments.

The Yunnan snub-nosed monkey (*Rhinopithecus bieti*) is a suitable model species to investigate the determinants of harem size. They live in polygynous one-male units (OMUs) or ‘harems’ that are part of a larger multilevel society [29,53]. They are characterized by strictly seasonal reproduction and a mean interbirth interval of approximately 2 years [54]. Harem holders possess nearly exclusive mating rights over all females in the harem. Females mate with harem holders many times during the mating season, but a very small number of females will mate surreptitiously with males outside their harem. The harem of the Yunnan snub-nosed monkey has a loose matrilineal relationship, and many of the co-resident females are related [29]. The number of adult females in Yunnan snub-nosed monkey harems varies and changes dynamically, and females can also transfer between harems [29]. Harem size is positively related to the quality of harem holders; i.e., low-quality males (such as older males or males with a long tenure) are easily rejected by females and have fewer females [29].

In this paper, we discuss the determinants of harem size variation and its underlying socioecological determinants in Yunnan snub-nosed monkeys. Sexual selection theory holds that females prefer high-quality males and that high-quality males are superior in inter-male competition, which leads to them having a larger harem size. Then, based on eleven years of observations, why has variation in the harem size of Yunnan snub-nosed monkeys been in a dynamic equilibrium, and why do lower-quality males still possess females (even a single female) for extended periods of time? Why do these females not abandon the lower-quality males and transfer into harems with higher-quality males? Here, we test the following hypotheses: (1) the reproductive success of harem holders increases while that of females decreases with an increase in harem size. (2) In larger harems, females will have to devote a greater portion of their time budget to grooming other females. Correspondingly, females receive more grooming from other females in larger harems. Harem holders decrease their grooming effort and receive more grooming as harem size increases.

## 2. Materials and Methods

### 2.1. Study Area and Study Group

This study was carried out at Xiangguqing in the Baimaxueshan Nature Reserve, Yunnan Province, China. The study area is characterized by a monsoon climate, with strong seasonal fluctuations in temperature and precipitation. The research area encompasses deciduous broadleaf forest, evergreen broadleaf forest, mixed deciduous broadleaf and conifer forest, evergreen conifer forest, and scrubland at an elevation of 2218–3417 m. Yunnan snub-nosed monkeys naturally subsist on lichens as well as leaves, buds, and fruits of angiosperms. Additional foods (including lichens, carrots, apples, peanuts, and lacquer tree fruits) were provided twice daily (around 9:00 and 17:00 h) by reserve staff as part of a tourism program [55]. Yunnan snub-nosed monkeys form large, multilevel groups (bands) consisting of multiple harems and a loosely attached all-male unit (AMU) [53]. The research group is a habituated wild group, having been separated from the local natural group since May 2008 [56]. All individuals were identifiable based on distinctive physical characteristics such as body size, hair pattern, scars, facial features, and pelage color [29]. From March 2010 to December 2020, a total of 26 OMUs and 1 AMU (182 individuals) were recorded in the research group. Each year, the research group consisted of 5–10 OMUs and 1 AMU, ranging from 45 to 72 individuals [54].

### 2.2. Data Collection

#### 2.2.1. Harem Composition and Reproductive Data

Data for this study were collected from 2010 to 2020 over a period of 2952 h. The observation time for each OMU is shown in Table 1. The present dataset is an extension of that presented in W Xia et al. [53]. The size and composition of all OMUs was recorded once per week. Data on birth events were recorded on an all-occurrence basis. Annual birth events occurred only during a circumscribed period lasting from February to June. The death of an infant within one year of being born was considered a reproductive failure.

#### 2.2.2. Grooming Data

From March 2013 to December 2018, data on grooming were collected from a total of 10 OMUs. Unstable OMUs (i.e., OMUs which disintegrated immediately after their establishment, generally <3 months) were excluded from data analysis. A total of 1723 h of grooming data were obtained. To ensure that observations continued over multiple full annual cycles, several observers were involved in data collection: D.Z., X.Y., and W.X. collected data from March 2013 to September 2014, and W.X. collected data from September 2014 to September 2016 and September 2016 to December 2018.

In order to ensure the accuracy of individual grooming data, before recording, all observers were familiar with the identification characteristics of each individual. On any given observation day, we stayed with the study subjects from 9:00 to 18:00 and collected grooming data on individuals in all visible OMUs. The criteria for selecting a focal OMU were: being in close proximity to the observer, being clearly visible, and not having any missing OMU members. To ensure balanced and comprehensive data collection, every effort was made to collect data on each OMU at least twice a month. All-occurrence recording was used to record male–female and female–female grooming bouts. For each grooming bout, the identity of the grooming initiator and recipient, and the duration of the grooming bout was recorded. The grooming time for each harem holder and female is shown in Table 1.

### 2.3. Data Analysis

The reproductive success of individuals in harems of different sizes was calculated by year. In general, a female’s reproductive success was measured as the number of offspring surviving to sexual maturity. Other criteria can be established based on research needs and the study duration [57]. In our study, we chose the survival of offspring beyond one year of life as the criterion for female reproductive success, because this is when infants are weaned and adult females prepare for their next reproductive event. The reproductive success of harem holders was the number of surviving infants in the harem. The time each OMU was observed was affected by weather and topography, resulting in variation in daily observation time and lack of data on some OMUs on particular observation days. We therefore conducted the grooming time analyses for each OMU using month as the unit of analysis. We first calculated the individual grooming time per month and then obtained an average across individuals for each OMU. The following variables pertaining to grooming were distinguished: grooming given (GG, grooming given/observation time), grooming received (GR, grooming received/observation time), and the ratio of grooming received to given (RGRG, time of grooming given/time of grooming received). We used Pearson’s correlation to test the magnitude of the correlation between harem size, reproductive success, and grooming. Curve estimation (linear, quadratic, power, and growth) was used to explore the relationship between harem size, reproductive success, and grooming. All statistical analyses were executed using SPSS25.0 and R v4.0.2 (R Core Team 2019). Adobe Illustrator CC 2019 was used to draw the figures.

## 3. Results

### Reproductive Success

**Figure 1:**
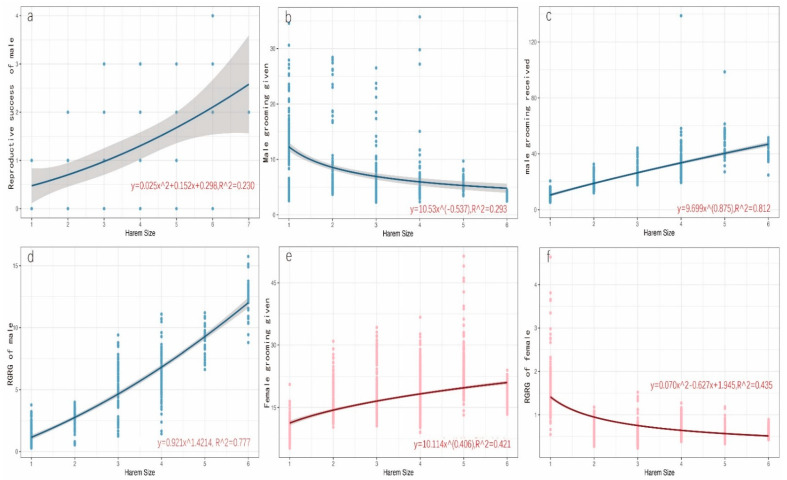
Relationships between harem size and reproductive success as well as grooming given, received, and the ratio of grooming received to given. (**a**) Reproductive success of harem holder and harem size, (**b**) Grooming given by harem holders and harem size, (**c**) Grooming received by harem holders and harem size, (**d**) The ratio of grooming received to given for harem holders and harem size, (**e**) Grooming given by females and harem size, and (**f**) The ratio of grooming received to given (RGRG) for females and harem size.

A total of 106 births were recorded during the study period, of which 91 infants survived. The reproductive success of harem holders and females as a function of harem size is shown in Table 2. Curve estimation analysis showed that the males’ reproductive success did not increase linearly with harem size but that a quadratic model fitting curve was the best (Reproductive success_male_ = 0.297 + 0.152 × harem size + 0.025 × harem size^2^, R^2^ = 0.230, F = 12.709, *p* < 0.001; Table S1, Figure 1a). Variation in harem size had no significant effect on female reproductive success (Table S2).

For harem holders, the amount of grooming given (GG) decreased with harem size; the optimal equation is GG_male_ = 10.53 × harem size^−0.54^ (R^2^ = 0.293, F = 204.667, *p* < 0.001; Table S3, Figure 1b). Grooming received (GR) and the ratio of grooming received to given (RGRG) increased with harem size; the optimal equations are GR_male_ = 9.699 × harem size^0.875^ (R^2^ = 0.812, F = 2131.774, *p* < 0.001; Table S4, Figure 1c); RGRG_male_ = 0.921 × harem size ^1.412^ (R^2^ = 0.777, F = 1723.570, *p* < 0.001; Table S5, Figure 1d). Males of single-female harems groomed significantly more (12.01 ± 6.04) and were groomed significantly less (9.97 ± 2.98) than males of multi-female harems (GG: 6.78 ± 5.64, F = 6.06, *p* < 0.001; GR: 30.49 ± 13.35, F = 126.88, *p* < 0.001). The RGRG of single-female harem holders (1.11 ± 0.74) was less than that of males of multi-female harems (6.10 ± 3.36, F = 215.51, *p* < 0.001).

Female grooming given (directed at both harem holder and other females) increased with harem size (GG_female_ = 10.114 × harem size^0.406^; R^2^ = 0.421, F = 962.773, *p* < 0.001; Table S6, Figure 1e). However, female grooming received did not increase with harem size (Table S7). The ratio of grooming received to given for females decreased with increasing harem size, with the optimal equation being: RGRG_female_ = 0.070 × harem size^2^ − 0.627 × harem size + 1.945 (R^2^ = 0.435, F = 508.534, *p* < 0.001; Table S8, Figure 1f).

By further dissecting female grooming behaviour, we found that females spent more time grooming other females when the harem size increased (GG_to other females_ = −235.13 + 264.04 × harem size − 25.81 × harem size^2^; R^2^ = 0.637, F = 1162.20, *p* < 0.001, Table S9). However, female grooming directed at harem holders did not show a fitting relationship with the harem size (Table S10). In terms of grooming received, females received less grooming from harem holders when the harem size increased (GR_from harem holders_ = 450.71 × harem size^−1.72^*,* R^2^ = 0.767, F = 4352.99, *p* < 0.001, Table S11). However, females were groomed for longer periods by other females when the harem size increased (GR_from other females_ = −241.18 + 264.29 × harem size − 25.93 harem size^2^*,* R^2^ = 0.652, F = 1236.81, *p* < 0.001, Table S12). The ratio of female grooming received by harem holders to female grooming given to harem holders was lower in larger harems (RGRG_from harem holders_ = 2.344 − 0.959 × harem size + 0.1 × harem size^2^; R^2^ = 0.596, F = 977.103, *p* < 0.001, Table S13). The ratio of female grooming received by other females to female grooming given to other females, however, was not strongly related to the harem size (Table S14).

## 4. Discussion

Our study documented the longitudinal stability in the distribution of harem sizes in a multilevel society of Yunnan snub-nosed monkeys, with an upper limit of seven adult females. These demographic properties are the outcome of male and female social investment decisions that ultimately determine their reproductive performance and success.

Our data support the hypothesis that males accumulate reproductive benefits from increased recruitment and monopolization of females. In our study group of Yunnan snub-nosed monkeys, harem size and male reproductive success were positively associated. Greater reproductive success is the ultimate consequence of harem size expansion. More proximately, the benefits that males derive from access to an increased number of females is greater grooming received (GR) and a greater ratio of grooming received to given (RGRG). This greater amount of grooming received did not necessitate greater grooming effort on the part of the male. The finding that male grooming effort is unaffected by harem size is contrary to Dunbar’s (1991) contention that larger groups require greater grooming effort. In fact, male grooming investment peaked in single-female harems (i.e., monogamous units). For the male in such units, investment in grooming is critical for retaining the only female in the harem and preventing the unit from disintegration, an outcome that would be detrimental to male reproductive success. In a multilevel setting where the risk of mate poaching is omnipresent, males of single-female harems are under particular pressure to put effort into holding on to their female.

Contrary to our expectation, female reproductive success was not the factor limiting harem size; the reproductive success of females did not decrease with increasing harem size. There may be several reasons for this finding. First, female Yunnan snub-nosed monkeys give birth every two years on average [54], thus reducing reproductive competition during years when they take a reproductive time out. Second, Yunnan snub-nosed monkey harems are composed mostly of related females [29], and nepotistic cooperation such as allocare and allonursing may promote successful reproduction. Third, extra-pair copulations may also reduce female reproductive pressure within their residential units [58,59,60]; fourth, food provisioning may replenish energy deficits and reduce food competition, thus alleviating the fecundity-depressing and mortality-increasing effects of group size to a certain extent.

Following Dunbar [32], individuals in larger groups need to devote more time to social grooming to maintain the cohesion of their groups. We found that female grooming given increased with an increase in harem size. In a polygynous context, an increase in the number of co-resident females may lead to intensified female intra-sexual competition for access to the single reproductive male. For example, intrasexual competition among females for sexual access to the harem leader has been shown to exist in golden snub-nosed monkeys (*Rhinopithecus roxellana*) where females in larger harems have less solicitation and mating success than females in smaller harems [61]. One possible solution for females to succeed in intra-sexual competition is to strengthen their social investments into the harem holder. However, when separating out female grooming given to males and other females, females showed an increased investment only with respect to other females. This result suggests that strengthening female–female bonds through grooming may be one of the key factors affecting harem size. As mentioned above, Yunnan snub-nosed monkeys give birth every two years on average [54], and the number of co-resident females in a harem is limited (the current record of co-resident females is less than seven). Overall, the pressure of reproductive competition was not great, thus obviating the need for excessive social investments into the harem holder. A more important challenge for females is to maintain strong bonds with other females. Female grooming investments in larger harems may help attenuate reproductive competition, increase tolerance around resources, set up in-kind reciprocal exchanges, and facilitate the care of the offspring by other females.

One potential limitation of our study design is that the reproductive success of harem holders was approximated by the number of surviving infants in the harem. The latter does not account for the possibility of harem holders siring infants in neighboring harems [62,63], as has been documented for the closely related golden snub-nosed monkey [62,63]. Currently, there are no data on the frequency of extra-harem paternities in Yunnan snub-nosed monkeys to assess the existence/extent of this limitation.

## 5. Conclusions

In conclusion, our analysis of the determinants of harem size in Yunnan snub-nosed monkeys revealed conflicting optima for the two sexes: the males’ reproductive success was positively correlated with harem size. For females, however, dwindling social benefits and excessive grooming investments appear to set an upper limit on harem enlargement. We also showed that males of monogamous units directed greater grooming effort at the female in order to prevent unit dissolution and loss of mating privileges.

## Figures and Tables

**Table 1:** Observation h and time spent grooming stratified by harem.

OMU	Observation Time for Different Harem Female Sizes (Months)						Observation Time (h)	Grooming Time (h)	Grooming Time of Each Male and Female (h)						
	1	2	3	4	5	6			Male	Female_1_	Female_2_	Female_3_	Female_4_	Female_5_	Female_6_
DGZ		29	34				336.52	130.3	13.69	42.41	47.32	26.87	/	/	/
SX				34	3		307.05	207.3	13.57	48.81	52.98	6.18	44.96	40.78	/
HL		21		28	12	1	365.91	297.1	18.52	14.6	51.35	35.34	55.09	62.66	59.49
DB	39		9	9	6		303.71	172.5	38.71	44.37	28.59	11.43	22.16	27.19	/
LHG	49	14					357.07	87.01	41.67	34.21	11.13	/	/	/	/
DS	44			10			38.65	90.46	14.42	13.86	13.28	35.81	13.09	/	/
HD			2	4	10	37	305.9	474.7	18.22	97.16	92.81	19.4	90.19	90.46	66.42
LB	35		6	2			536.8	93.49	43.1	27.72	11.21	11.46	/	/	/
ML			23	3			273.79	99.33	9.81	28.7	28.14	28.66	4.02	/	/
JG	9	15	2				126.22	71.1	32.47	10.58	9.05	19	/	/	/
All	176	81	74	91	31	37	2951.63	1723.12	244.18						

The abbreviations in the first row of the table indicate the name of OMU.

**Table 2:** Reproductive success (i.e., number of surviving infants) of harem holders and females in harems of different size.

Harem Female Size	Successful Reproductive Events	Mean Reproductive Success of Harem Holder	Mean Reproductive Success of Females
1	10	0.435 ± 0.507, *n* = 23	0.435 ± 0.507, *n* = 23
2	12	0.857 ± 0.663, *n* = 14	0.429 ± 0.504, *n* = 28
3	12	0.857 ± 0.949, *n* = 14	0.286 ± 0.457, *n* = 42
4	33	1.375 ± 1.135, *n* = 24	0.344 ± 0.477, *n* = 96
5	7	1.167 ± 1.169, *n* = 6	0.233 ± 0.430, *n* = 30
6	15	2.5 ± 1.517, *n* = 6	0.417 ± 0.500, *n* = 36
7	2	2 ± /, *n* = 1	0.286 ± 0.488, *n* = 7

## Data Availability

Not applicable.

## Supplementary Materials

The following are available online at https://www.mdpi.com/article/10.3390/ani11102915/s1, Table S1: Results of regression of harem size against male reproductive success, Table S2: Results of regression of harem size against female reproductive success, Table S3: Results of regression of harem size against male grooming given, Table S4: Results of regression of harem size against male grooming received, Table S5: Results of regression of harem size against the ratio of male grooming received to given, Table S6: Results of regression of harem size against female grooming given, Table S7: Results of regression of harem size against female grooming received, Table S8: Results of regression of harem size against the ratio of female grooming received to given, Table S9: Results of regression of harem size against the female grooming other females, Table S10: Results of regression of harem size against the female grooming harem holders, Table S11: Results of regression of harem size against the female grooming received from harem holders, Table S12: Results of regression of harem size against the female grooming received from other females, Table S13: Results of regression of harem size against the ratio of female grooming received to given (RGRG) from harem holders, Table S14: Results of regression of harem size against the ratio of female grooming received to given (RGRG) from other females.
