# Supplementary material for: Determinants of Harem Size in a Polygynous Primate: Reproductive Success and Social Benefits
Source: Animals (Basel). 2021 Oct 9;11(10):2915. doi: 10.3390/ani11102915 (PMC8532613; doi:10.3390/ani11102915)
Supplement: Supplementary file 1 [file animals-11-02915-s001.zip › animals-1364089-supplementary.pdf]

Table S1. Results of regression of harem size against male reproductive success.

	R^2	F	$df1$	$df2$	p	AIC
Linear	0.226	25.145	1	86	< 0.001	76.976
**Quadratic**	**0.230**	**12.709**	**2**	**85**	**< 0.001**	**76.916**

Table S2. Results of regression of harem size against female reproductive success.

	R^2	F	$df1$	$df2$	p	AIC
Linear	0.012	1.075	1	86	0.303	13.081
Quadratic	0.019	0.836	2	85	0.437	15.003

Table S3. Results of regression of harem size against male grooming given.

	R^2	F	df_1	df_2	p	AIC
Linear	0.210	131.194	1	495	< 0.001	15562.727
Quadratic	0.216	68.089	2	494	< 0.001	15435.105
**Power**	**0.293**	**204.667**	**1**	**495**	**< 0.001**	**147.827**
Growth	0.289	201.562	1	495	< 0.001	148.477

Table S4. Results of regression of harem size against male grooming received.

	R^2	F	df_1	df_2	p	AIC
Linear	0.656	941.878	1	495	< 0.001	36660.102
Quadratic	0.670	500.821	2	494	< 0.001	35150.683
**Power**	**0.812**	**2131.774**	**1**	**495**	**< 0.001**	**39.119**
Growth	0.744	1439.454	1	495	< 0.001	52.403

Table S5. Results of regression of harem size against the ratio of male grooming received to given.

	R^2	F	df_1	df_2	p	AIC
Linear	0.832	2455.441	1	495	< 0.001	1098.89
Quadratic	0.841	1305.428	2	494	< 0.001	1044.232
**Power**	**0.777**	**1723.570**	**1**	**495**	**< 0.001**	**121.618**
Growth	0.733	1362.053	1	495	< 0.001	144.904

Table S6. Results of regression of harem size against female grooming given.

	R^2	F	df_1	df_2	p	AIC
Linear	0.221	376.348	1	1324	< 0.001	35301.386
Quadratic	0.323	314.956	2	1323	< 0.001	30715.003
**Power**	**0.421**	**962.773**	**1**	**1324**	**< 0.001**	**95.434**
Growth	0.314	606.943	1	1324	< 0.001	112.652

Table S7. Results of regression of harem size against female grooming received.

	R^2	F	df_1	df_2	p	AIC
Linear	0.00021	0.278	1	1324	0.598	24604.150
Quadratic	0.003	2.187	2	1323	0.113	24530.217
Power	0.0004	0.467	1	1324	0.495	154.038
Growth	0.001	0.349	1	1324	0.246	153.937

Table S8. Results of regression of harem size against the ratio of female grooming received to given.

	R^2	F	df_1	df_2	p	AIC
Linear	0.248	436.781	1	1324	< 0.001	173.853
**Quadratic**	**0.435**	**508.534**	**2**	**1323**	**< 0.001**	**133.213**
Power	0.348	707.694	1	1324	< 0.001	136.353
Growth	0.220	372.583	1	1324	< 0.001	162.89

Table S9. Results of regression of harem size against the female grooming other females.

	R^2	F	df_1	df_2	p	AIC
Linear	0.504	1347.394	1	1324	< 0.001	21566505.81
**Quadratic**	**0.637**	**1162.196**	**2**	**1323**	**< 0.001**	15783641.87

Table S10. Results of regression of harem size against the female grooming harem holders.

	R^2	F	df_1	df_2	p	AIC
Linear	0.081	116.019	1	1324	< 0.001	17408195.19
Quadratic	0.086	62.111	2	1323	< 0.001	17308483.11
Power	0.069	97.895	1	1324	< 0.001	223.758
Growth	0.086	124.316	1	1324	< 0.001	219.713

Table S11. Results of regression of harem size against the female grooming received from harem holders.

	R^2	F	df_1	df_2	p	AIC
Linear	0.472	1183.253	1	1324	< 0.001	23426062.94
Quadratic	0.661	1289.130	2	1323	< 0.001	15044041.71
**Power**	**0.767**	**1212.519**	**1**	**1324**	**< 0.001**	**370.798**
Growth	0.701	3110.784	1	1324	< 0.001	474.100

Table S12. Results of regression of harem size against the female grooming received from other females.

	R^2	F	df_1	df_2	p	AIC
Linear	0.513	1395.290	1	1324	< 0.001	20520277.28
**Quadratic**	**0.652**	**1236.805**	**2**	**1323**	**< 0.001**	**14686381.09**

Table S13. Results of regression of harem size against the ratio of female grooming received to given (RGRG) from harem holders.

	R^2	F	df_1	df_2	p	AIC
Linear	0.424	972.928	1	1324	< 0.001	290.594
**Quadratic**	**0.596**	**977.103**	**2**	**1323**	**< 0.001**	**206.117**
Power	0.697	3045.150	1	1324	< 0.001	413.825
Growth	0.608	2054.898	1	1324	< 0.001	534.518

Table S14. Results of regression of harem size against the ratio of female grooming received to given (RGRG) from other females.

	R^2	F	df_1	df_2	p	AIC
Linear	0.004	4.082	1	1324	0.044	149.445
Quadratic	0.004	2.403	2	1323	0.091	151.351
Power	0.001	0.068	1	1324	0.408	97.923
Growth	0.001	0.255	1	1324	0.614	97.960
